# Revised whole genome and DNA methylome of *Mycobacterium marinum* type strain ATCC 927^T^

**DOI:** 10.1128/mra.01016-23

**Published:** 2024-02-28

**Authors:** Kirsi Savijoki, Paulina Deptula, Richard J. Roberts, Milka Hammarén, Jari Yli-Kauhaluoma, Pekka Varmanen, Mataleena Parikka

**Affiliations:** 1 Drug Research Program, Division of Pharmaceutical Chemistry and Technology, Faculty of Pharmacy, University of Helsinki, Helsinki, Finland; 2 Faculty of Agriculture and Forestry, University of Helsinki, Helsinki, Finland; 3 Department of Food Science (FOOD), University of Copenhagen, Frederiksberg, Denmark; 4 New England Biolabs, Ipswich, Massachusetts, USA; 5 Faculty of Medicine and Health Technology, Tampere University, Tampere, Finland; 6 European Molecular Biology Laboratory, Heidelberg, Germany; Portland State University, Portland, Oregon, USA

**Keywords:** *Mycobacterium marinum*, tuberculosis, SMRT-PacBio, genome analysis, plasmid, DNA methylome

## Abstract

*Mycobacterium marinum*, a slow-growing Actinobacterium, typically induces tuberculosis-like disease in fish. Here, we report a new reference sequence for *M. marinum* ATCC 927^T^, along with its DNA methylome. This aims to maximize the research potential of this type strain and facilitates investigations into the pathomechanisms of human tuberculosis.

## ANNOUNCEMENT

*Mycobacterium marinum* (Mmr) strain ATCC 927^T^, a slow-growing Gram-positive bacterium, was originally isolated from spontaneous tuberculosis (TB) in fish (1, 2). Phylogenetic analysis based on 16S rRNA gene sequences revealed 99.3% identity to *Mycobacterium tuberculosis* (Mtb), which is the leading etiological agent of TB in humans (3, 4). Like Mtb, Mmr also survives inside its natural host (zebrafish) and triggers a positive tuberculin test during infection (5–10), making it an excellent surrogate model for Mtb.

**Fig 1:**
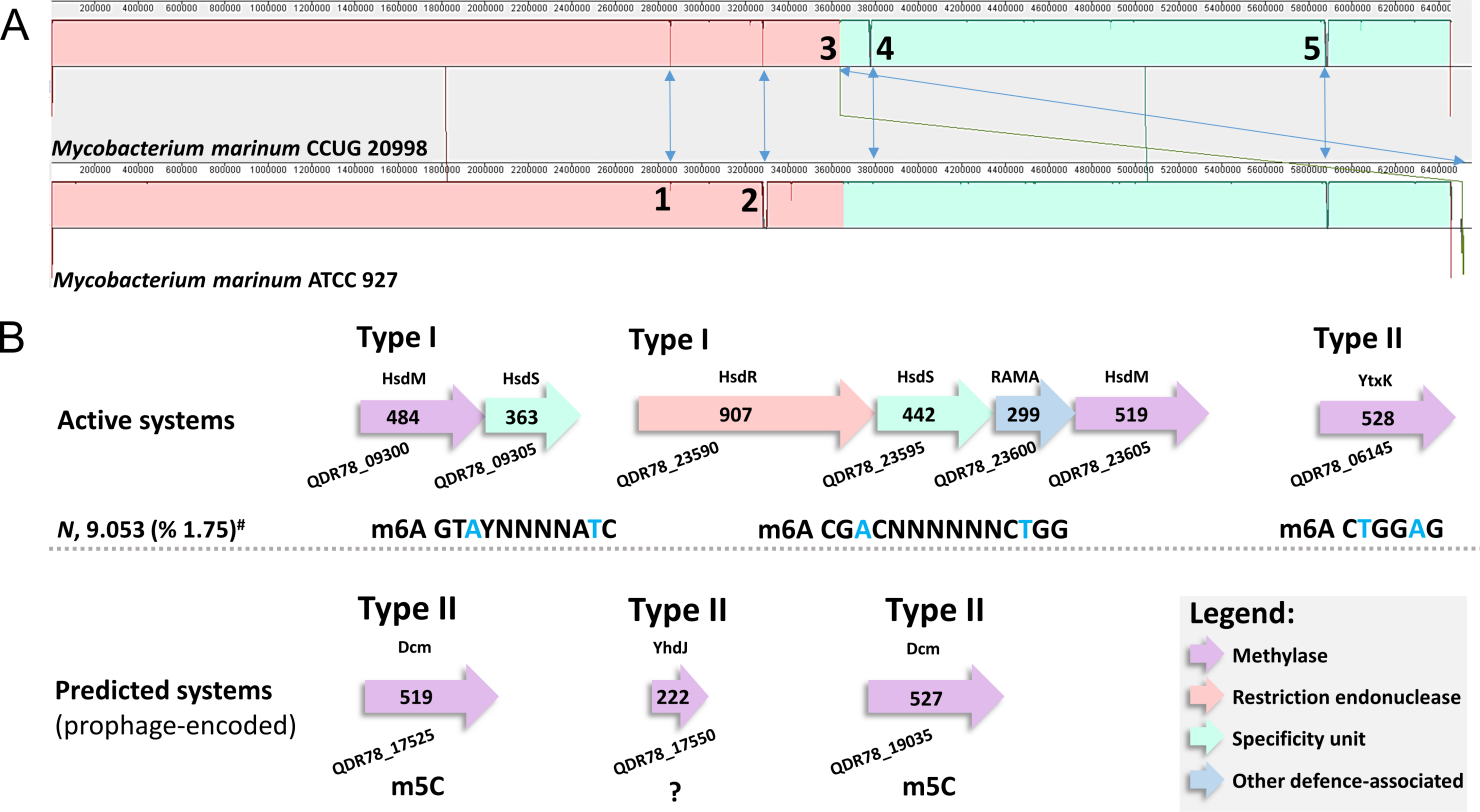
(**A**) Whole genome alignment of strain ATCC 927 to its previously sequenced passage variant CCUG 20998. ProgressiveMauve (27) was used to align the indicated genomes in a step-by-step manner for the identification of conserved regions and rearrangements between the two genomes. Significant differences are indicated with arrows and numbered as follows. 1, a truncated ABC transporter permease (QDR78_11875 versus intact CCUG20998_RS12005) due to a sequence deletion, 2, a truncated PE family protein due to a frameshift (QDR78_13350 versus CCUG20998_RS28445); pseudogenes on both genomes. 3, an insertion of six genes (CCUG20998_RS15230–CCUG20998_RS15255) coding for an IS3 family transposase, recombinase (a pseudogene), cytochrome P450, TetR/AcrR family transcriptional regulator, a frameshifted TetR/AcrR family transcriptional regulator, and a frameshifted IS256 family transposase. These genes correspond to plasmid-located genes in ATCC 927^T^ (QDR78_27190–QDR78_27215) coding for TetR/AcrR family transcriptional regulator, TetR/AcrR family transcriptional regulator, cytochrome P450, DUF308 domain-containing protein (a membrane-associated protein), a pseudogene recombinase family protein, and an IS3 family transposase, respectively. 4, a truncated non-ribosomal peptide synthase/polyketide synthase, which is also split into two parts: CCUG20998_RS28465 (AMP-binding protein, 322 amino acids) and CCUG20998_RS28470 (amino acid adenylation domain-containing protein, 1,468 amino acids), versus QDR78_13590 (9,908 amino acids). 5, another non-ribosomal peptide synthase/polyketide synthase: CCUG20998_RS15760 (12,024 amino acids) versus QDR78_15505 (9,880 amino acids). (**B**) Active and predicted R-M systems found in strain ATCC 927^T^, along with matched methylation motifs detected through SMRT whole methylome analysis. The motifs GTAYNNNNATC and CTGGAG and their corresponding R-M systems are also found in the *Mycobacterium marinum* strain MMA1 (REBASE org. #46029). Other PacBio-sequenced strains contain either the first (strain 050012) or the second (*Mycobacterium marinum* strains E11 and H01) of the R-M system-motif pairs, whereas the R-M system with recognition motif CGACNNNNNNCTGG is unique, with modified adenines (indicated as T for complementary strand) marked in blue. Strain MMA1, a human-infection-associated isolate (26), also possesses an additional Type I system with recognition motif CCACNNNNNNNTCCC that is not found in other strains. No activity was detected for the prophage-encoded methyltransferases. #, numbers of detected m6A type modifications (%, number of detected m6A modifications per total number of all detected modifications).

We revised the whole-genome sequence of ATCC 927^T^ using PacBio SMRT, identified the methylation motifs, and matched them to corresponding restriction-modification (R-M) systems. Freeze-dried ATCC 927^T^, from the American Type Culture Collection, was cultured in Middlebrook 7H9 broth containing 10% Albumin−Dextrose−Catalase (Thermo Fisher Scientific) and 0.5% glycerol for 7 days at 29°C. The genomic DNA was extracted using the MagAttract HMW DNA Kit (Qiagen, Nordic). Library preparation, quality control, sequencing, and assembly were performed by Novogene (UK) using their standard operating procedures. PacBio libraries were prepared using the SMRTbell Express Template Prep Kit 2.0 (Pacific Biosciences), following the standard PacBio protocol (11) and selecting DNA fragments > 17 kb using the BluePippin system (Sage Sciences). The libraries sequenced on a PacBio Sequel II instrument using CLR mode generated a total of 334,798 reads with an average length of 77,683 bp and an *N*_50_ value of 120,575  bp. Default parameters were used for all the following software unless otherwise specified. Base calling and demultiplexing were performed using SMRT Link V11.1 software (11). Canu-1.6 (12) and HGAP-4 (13) were used for *de novo* assembly of the PacBio long reads, incorporating pre-processing steps for Canu and as a separate step for HGAP. Alignment of pre-assembled reads, Arrow-2.3 (14), and Circlator-1.5.3 (15) were used for error correction, polishing, and circular assembly processes, respectively. The completeness of the assemblies was assessed using BUSCO 4.0.2 (16). The resulting two scaffolds with read depths of 499× for the chromosome and 309× for the plasmid were annotated at NCBI using the Prokaryotic Genome Annotation Pipeline PGAP-6.5 (17–19). Prophage sequences were predicted using the PHASTER server (20), and SEQWARE methylome analysis at REBASE (21) was conducted as previously described (22, 23). Due to the high sequence identity (99.63%) of the plasmid to the previously published plasmid sequence of ATCC 927^T^ (24), the plasmid was named pMMRN. Table 1 lists all central genomic data of ATCC 927^T^ in comparison to other related Mmr genomes. Figure 1 illustrates the major differences between ATCC 927^T^ and its passage variant CCUG 20998, also defined by PacBio SMRT (25), as well as indicates the methylation motifs with their corresponding active and predicted R-M systems. In summary, this study provides a new reference sequence for ATCC 927^T^ and reports the first in-depth DNA methylome analysis for this non-tuberculous bacterial model.

**TABLE 1:** Comparison of the whole genome details of the Mmr ATCC 927^T^ sequenced here with the same strain sequenced earlier by Nanopore/Illumina (NI) (25) and its passage variant CCUG 20998 using PacBio SMRT (PB) (26)^*e*^

	Genome							Plasmid					Origin
Name of the strain	Accession no.	Size (bp)	G + C (%)	No. Genes	No. CDSs	No. pgenes^*a*^	No. RNAs	Accession no.	Size (bp)	No. Genes	No. tRNAs	G + C (%)	
ATCC 927^T^ (NI)	NZ_AP018496.1	6,451,936	65.5	5,744	5,150	537	57	NZ_AP018497.1	127,402^*d*^	130	1	63.50	25
CCUG 20998 (PB)	NZ_CP024190.1	6,453,310	65.7	5,411	5,203	152	56	NR^*b*^	127,576^*c*^	112^*c*^	1^*c*^	63.96^*c*^	26
ATCC 927^T^ (PB)	CP129323	6,455,215	65.7	5,502	5,300	145	57	CP129324	127,576	112	1	63.96	This study

^*a*^ pgenes, pseudogenes.

^*b*^ NR, not reported.

^*c*^ Due to the lack of plasmid sequence at NCBI, the annotation was not updated from the original.

^*d*^ The difference in length is a result of the accumulation of sequencing errors, particularly in regions of homopolymeric stretches, as determined by BLASTN 2.15.0 + nucleotide alignment.

^*e*^ For an accurate comparison, all previously generated whole-genome sequences were obtained from the RefSeq database, where they were subjected to PGAP re-annotation.

## Data Availability

The ATCC 927^T^ genome and plasmid pMMRN sequences are available at NCBI with accession numbers CP129323 and CP129324, respectively. The DNA methylome analysis is available at REBASE with the Org# number 27274. The BioProject and SRA database accession numbers are PRJNA955429 and SRX22236853.

## AFTER EPUB

*[This article was published on 28 February 2024 with some author given names and surnames reversed in the byline. The byline was updated in the current version, posted on 4 March 2024.]*
